# Finding Sequences for over 270 Orphan Enzymes

**DOI:** 10.1371/journal.pone.0097250

**Published:** 2014-05-14

**Authors:** Alexander G. Shearer, Tomer Altman, Christine D. Rhee

**Affiliations:** 1 Clover Collective, Mountain View, California, United States of America; 2 Stanford University, Stanford, California, United States of America; Université Paris-Sud, France

## Abstract

Despite advances in sequencing technology, there are still significant numbers of well-characterized enzymatic activities for which there are no known associated sequences. These ‘orphan enzymes’ represent glaring holes in our biological understanding, and it is a top priority to reunite them with their coding sequences. Here we report a methodology for resolving orphan enzymes through a combination of database search and literature review. Using this method we were able to reconnect over 270 orphan enzymes with their corresponding sequence. This success points toward how we can systematically eliminate the remaining orphan enzymes and prevent the introduction of future orphan enzymes.

## Introduction

Nucleotide or amino-acid sequence data is the *lingua franca* that connects disparate branches of modern biology. Confronted with a novel amino acid sequence with no known function, researchers search sequence databases such as the NCBI non-redundant protein sequences database for significant hits [1]. From these results they receive clues to protein function in the form of predicted binding sites, catalytic sites, structural motifs, protein family membership, and identification of highly similar characterized proteins. These functional annotations link sequence data to knowledge about enzyme function contained in databases or the literature. Researchers infer a wealth of knowledge about a protein's function based on the sequence data before a single lab experiment is performed.

**Figure 1:**
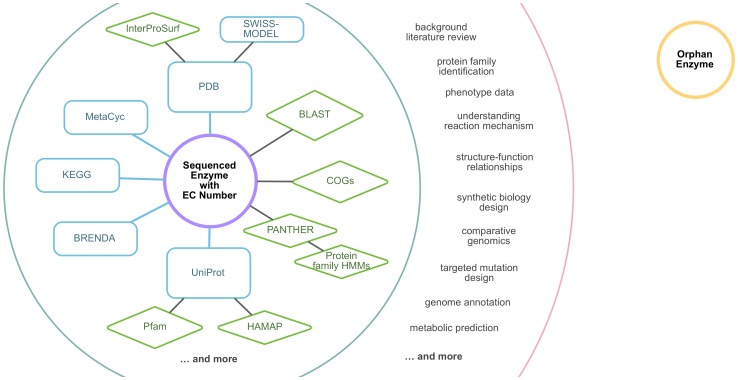
Orphan enzymes break the link between sequence and function. Amino acid sequence information connects knowledge about protein function in major databases (blue) and sequence-based predictive tools (green), enabling many critical tasks in contemporary biology (outer ring). The absence of sequence data for orphan enzymes that have been experimentally characterized (yellow) disconnects knowledge about those enzyme activities, their associated motifs, domains, and other sequence-linked traits from this family of databases and sequence-based predictive tools. This valuable information remains “trapped” in the literature, inaccessible for genome annotations, predictions, and to guide hypothesis formation for bench biology.

Orphan enzymes are those enzymes that have been experimentally characterized but lack associated amino acid sequences. This absence of sequence data for an orphan enzyme disconnects it from all knowledge derived from the sequence or the structure of the enzyme, such as its motifs, domains, and active sites (FIGURE 1).

**Figure 2:**
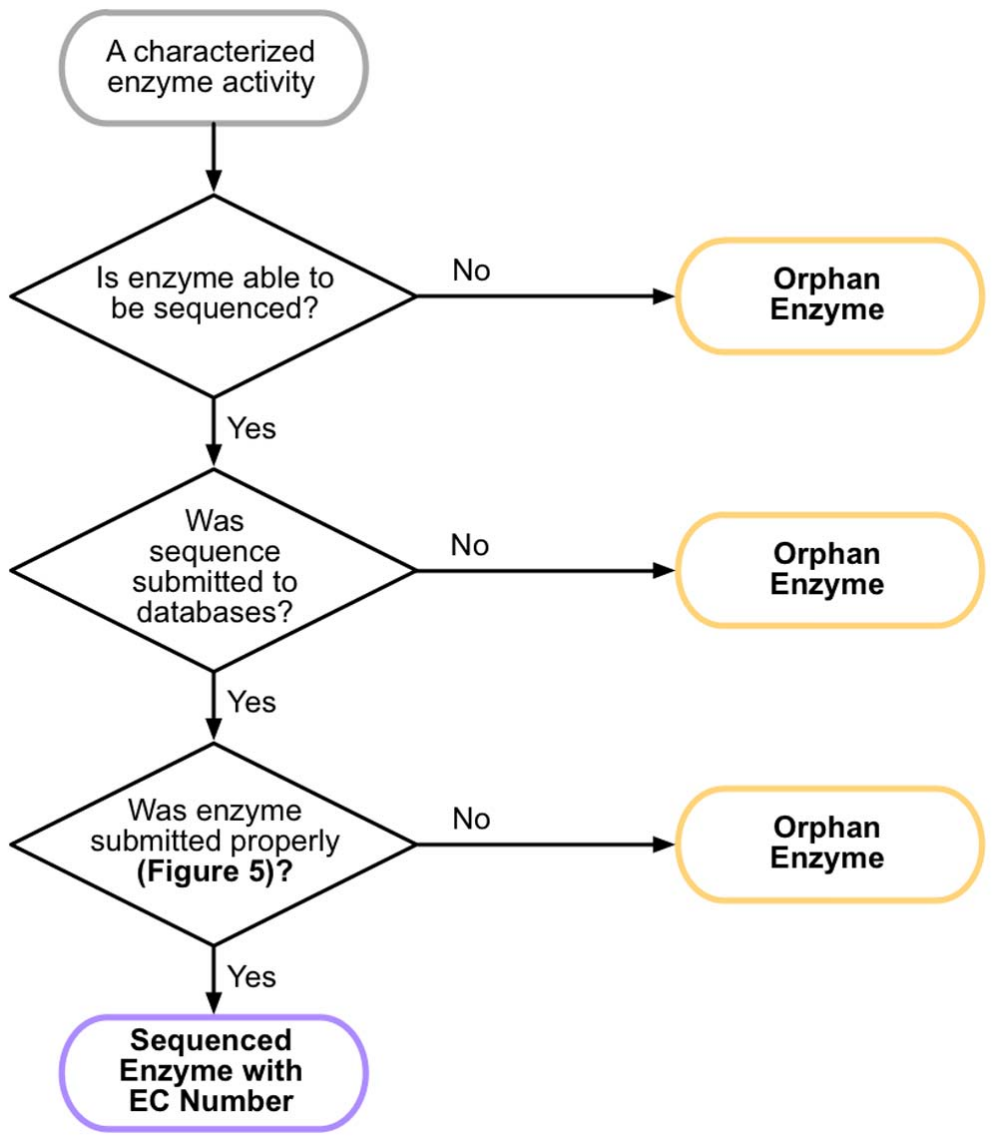
Three chokepoints generate orphan enzymes. Starting from an enzyme activity that has been characterized in the lab (gray), there are three major chokepoints that can lead to enzyme activity not being linked to sequence data, generating an orphan enzyme (yellow) rather than a sequenced enzyme with an associated EC number (purple). In the laboratory stage, an enzyme may not be sequenced due to issues such as complexity of the sequencing process for that enzyme, a loss of researcher interest, or inadequate funds to pursue sequencing. When an enzyme has been sequenced, sequence data may not be deposited in GenBank and other major sequence databases, despite the presence of sequence data in a scientific publication. Finally, errors in depositing the sequence with those databases can prevent connection of that sequence data with the enzyme activity (see FIGURE 5).

Orphan enzymes occur due to disruptions in the process between experimental characterization of an enzyme and proper annotation of its corresponding sequence in major sequence repositories (FIGURE 2). The most obvious source of disruption is whether the enzyme was sequenced in the first place. For older enzyme activities, the prohibitive nature of protein sequencing meant many activities were extensively characterized in the lab but never sequenced. In the era of high throughput sequencing, a failure to sequence an enzyme tends to occur only in exceptional cases (e.g. a membrane protein) where additional expertise is required to successfully sequence the isolated enzyme. It is also conceivable that some enzymes were sequenced, but that their sequence data was never deposited in major sequence repositories. Finally, issues with properly annotating the enzyme sequence may mean that a deposited sequence is not properly linked to its characterized enzyme activity.

The precise identification of orphan enzymes is predicated on an authoritative systematization of enzyme knowledge. The International Union of Biochemistry and Molecular Biology's Enzyme Commission curates the definitive controlled vocabulary of experimentally characterized enzymatic activities [2]. Each enzyme activity classified within the EC system is assigned a unique identifier consisting of four numbers (for example, EC # 1.1.1.3 is homoserine dehydrogenase, EC # 1.1.1.11 is D-arabinitol 4-dehydrogenase, EC # 1.1.1.12 is L-arabinitol 4-dehydrogenase, and EC # 2.1.1.45 is thymidylate synthase). The first three numbers in the identifier describe the location of the activity in the EC system's three-level hierarchy, whereas the last number is incremented for each subsequent entry.

The scale of the orphan enzyme problem is surprisingly large. When Lespinet, Labedan, and others first brought major attention to the issue of orphan enzymes, approximately 40% of the EC entries had no associated sequence data [3]–[5]. A subsequent analysis of the approximately 4,000 EC entries confirmed this lack of sequence data [6]. As a consequence, every sequence-based predictive tool is missing data from over a thousand experimentally characterized enzymes with distinctive functions and biological roles. This is both a dramatic cost in terms of “lost” research effort and a tremendous opportunity to recover a vast quantity of prior research and connect it to modern, sequenced-based methods.

Given the scale of the problem, it is critical to develop the most efficient method to identify sequences for as many orphan enzymes as possible. The three major approaches to address this problem, in order of increasing difficulty, are (1) by reviewing the relevant literature and sequence databases, (2) via computational prediction of candidate sequences, and (3) via purification, assay, and sequencing. A previous analysis of a small sample of orphan enzymes predicted that 80% of the current orphans may represent a true lack of sequence data, meaning that those enzymes were never sequenced [6]. In these cases, approaches (2) and (3) apply, although laboratory identification is sometimes impossible due to lost or hard-to-culture source organisms. Computational methods may fill that gap, using context-based techniques to identify candidate sequence for orphan enzymes [7]–[10]. The remaining 20% of orphan enzymes are predicted to actually have sequence data available, buried in papers and patents, or incorrectly annotated in sequence databases. This literature and database approach is obviously the most cost-effective way to find sequence data for orphan enzymes, as it involves no new experiments or experimental validation of computationally predicted candidate sequences. However, it was unclear whether the estimate that 20% of putative orphan enzymes would be identifiable using approach (1) would hold up when all the orphan enzymes were reviewed, what the best methods would be to examine the literature and databases for sequence data, and how many sequences this approach would actually yield.

In the current study we examined 1,122 orphan EC activities and identified sequence data via a combined literature, database, and patent search. We assessed which methods of searching these resources to identify enzyme sequence data were most efficient. We identified sequences for over 250 orphan enzymes by searching these resources. We also collected a wealth of identification information for the remaining orphan enzymes that will be essential for guiding future laboratory and computational efforts to find sequences for them. Based on our experience, we have also outlined a process that can be applied to evaluating the remaining orphan enzyme activities.

## Results

### 23% of EC enzyme activities were putative orphans

Prior surveys of orphan enzymes suggest that up to 40% of the enzymes classified within the EC system lack associated sequence data [6]. To be classified within the EC system, an enzyme activity must be experimentally characterized. This means that activities that are presumed to exist (e.g. missing steps in known metabolic pathways) but which have not yet been explicitly demonstrated in the lab are not eligible for an EC number (the full guidance for requesting an EC number assignment for an enzyme activity can be found at the EC website [11]. Enzyme activities classified within the EC system have typically been demonstrated experimentally via partial or complete purification of an enzyme, although in some cases activities were identified from raw cell extracts or similar unpurified sources. If the initial estimate of 40% of EC activities being orphans were still correct, that would represent nearly 2,000 experimentally characterized enzyme activities for which no sequence data were available.

We generated the initial list of putative orphan enzyme activities by reviewing major sequence databases (namely, UniProt, Enzyme, NCBI Entrez Protein, Orenza, and BioCyc) for sequence data for each enzyme activity listed within the EC system [12]–[16]. An enzyme activity was counted as an orphan only if it lacked sequence across all of the above databases.

1,122 putative orphans were identified from the set of 4,858 EC enzyme activities we evaluated. At 23% of the total EC activities, this was a significant decrease from the burden of orphans identified in previous studies. However, this meant that still nearly 1 in 4 EC activities we evaluated remained without sequence.

### Finding sequences for orphans via literature, patent, and database search

We next wanted to determine for how many of the 1,122 putative orphan activities we could identify sequence data from the literature, databases, and patents. A previous representative sampling determined that 20% of putative orphan enzymes were likely to have sequence data that could be found in sequence databases, and we hypothesized that the same rate would hold true for the entire set of EC orphan enzyme activities [6].

**Figure 3:**
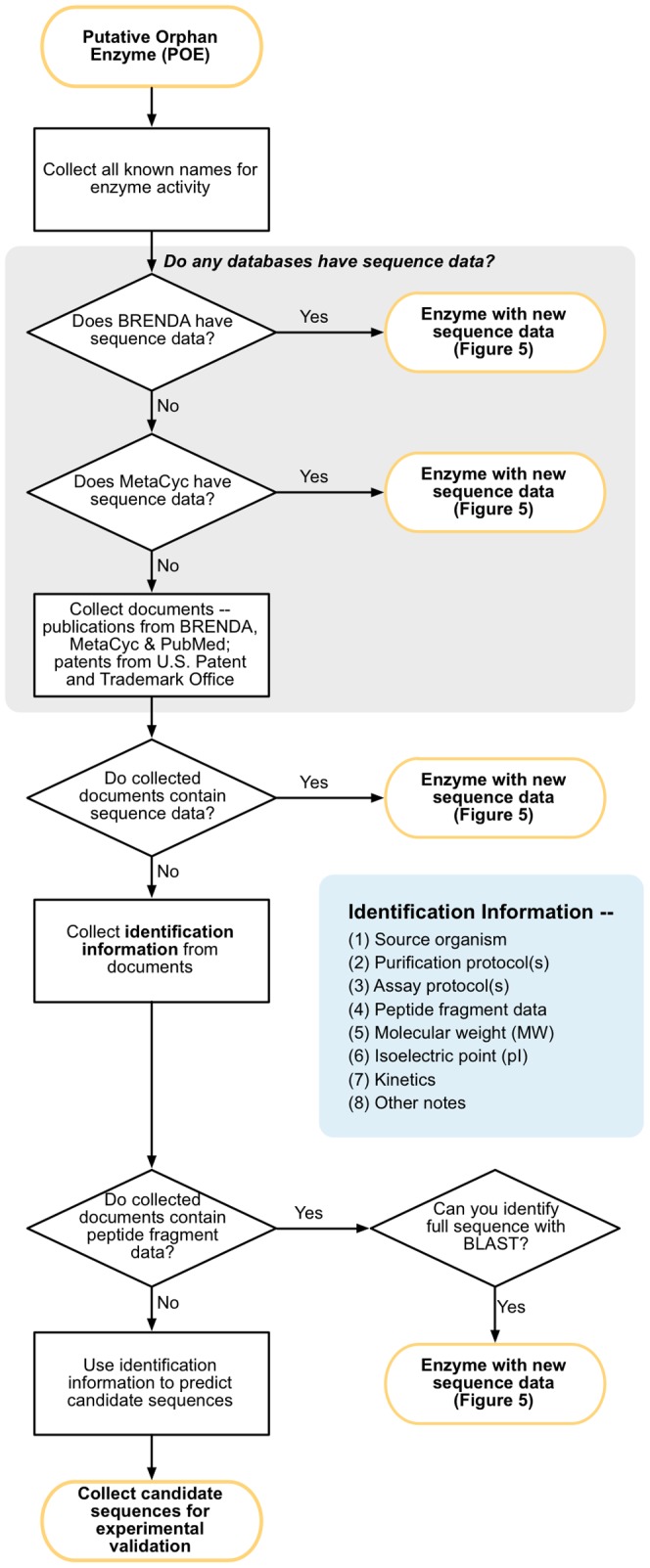
Putative orphan enzymes were evaluated via the literature and databases to find sequences or identification information. Each putative orphan was evaluated via a multistep process relying on sequence databases, the literature, and patent databases. Each evaluation process began by collecting all names for the enzyme activity. The BRENDA and MetaCyc databases, which link enzyme data to EC numbers, were then examined. At this and all subsequent steps, sequence data was collected when found. Documents were then collected, including texts cited in BRENDA and MetaCyc, texts found via PubMed search, and patents from the U.S. Patent and Trademark Office. Identification information (inset box) were collected from each publication. When available, peptide sequence data were collected to attempt to identify the full protein sequence via BLAST. When possible, identification information were used to predict candidate sequences for subsequent testing in the laboratory.

We evaluated each of the 1,122 putative orphan enzymes using a multi-step process of examining sequence databases, the scientific literature, and patents (FIGURE 3 and described more fully in Methods). We checked the BRENDA and MetaCyc databases using the list of terms associated with an EC activity (EC number, systematic, and common names) [14], [17]. These same terms were then used to search the literature. At each of these search stages, research papers were collected and reviewed for the presence of accession numbers, sequence data, or other identification information (described below). If no sequence was identified during the database and literature search steps, United States patents were then reviewed in a similar manner [18]. The full list of reviewed enzymes and their final status is included in TABLE S1.

As we believed that the majority of orphans would still need to be identified in the lab or via computational methods, we collected identification information for each orphan enzyme (FIGURE 3). Identification information includes traits that would potentially be useful for vetting computational predictions, such as molecular weight and isoelectric point. They also include pointers to resources that would likely assist in laboratory identifications, such as publications describing the enzyme's purification or assay protocols. The identification information for all the evaluated orphans are contained, along with the linked citations, in TABLE S2.

### Identification and analysis of 275 orphan enzymes

We identified amino acid sequence data for 275 orphan enzymes (TABLE 1). We were unable to find sequence data for the remaining 847 orphans, which we term “true” orphan enzymes.

**Table 1:** Sequences were identified for 275 putative orphan enzymes, most frequently by fixing database errors.

**A. Orphans with new sequence data from literature-based method**		
***Putative orphan enzymes (POEs)***	***1,122***	
**Orphan enzymes with new sequence data**	**275**	**24.51%**
Remaining orphan enzymes	847	75.49%
**B. Characterization of 275 identified orphan enzymes**		
***Missing annotation updates***		***49.09%***
Sequence annotated to class level (e.g. “aldehyde reductase”)	49	17.69%
Sequence needed addition of new enzyme activity to a properly annotated existing enzyme	36	13.00%
Sequence lacked assigned activities	29	10.47%
Sequence annotated with similar activity within same class as orphan activity	21	7.58%
***Data labeling errors***		***50.91%***
Sequence lacked EC number	109	39.35%
Sequence lacked synonymous names	28	10.11%
Sequence misannotated with incorrect EC number	3	1.08%
**C. How 275 orphan sequences were found**		
Literature and database review	268	97.45%
BLASTing with N-terminal sequence data	5	1.82%
Molecular weight data compared to sequenced genome	2	0.73%

Sequences were found for 275 putative orphan enzymes (25% of the total) by searching through the literature, sequence databases, and patents. Approximately half of the orphans for which sequences could be found were “annotation updates,” in which the sequence for the enzyme in major databases was annotated with no activity, with a less specific activity, with an incorrect activity that was in the same general class as the correct one, or with another *correct* activity that the enzyme also carries out. The remaining orphans fell into the “data inconsistency” category. Orphan enzymes in this category were in some way annotated to sequence data, but a lack of an EC number or a nomenclature mismatch meant that searching these databases for the activity did not yield any sequence data.

We further evaluated the 275 resolved orphan enzymes to discover where the disruption occurred between the experimental identification of sequences for these enzymes and their inclusion in major sequence databases with appropriate functional annotation.

Resolved orphans were split nearly evenly between cases of “annotation updates” and “data inconsistency.”

Annotation updates involved the sequence coding for the the enzyme being present in major sequence databases, but simply being out of date with respect to the full knowledge available about the enzyme following completion of our literature and database review. Since genome annotations in GenBank are primarily updated by their original submitters, it is not uncommon for an annotation to become incomplete relative to the full, available data over time. Approximately a third (49) of the annotation update cases involved enzymes that were already annotated correctly with a “class level” enzyme activity within the EC hierarchy with no additional specific activity (e.g. being annotated to “aldehyde dehydrogenase” when the actual activity was the more specific “fluoroacetaldehyde dehydrogenase”). 36 annotation updates were cases where the sequences were already correctly annotated with a different activity and the orphan enzyme activity was an additional reaction the enzyme was *also* capable of catalyzing. In 29 cases the orphan activity was the first enzyme activity of any kind assigned to the sequence. In 21 cases, the new activity replaced a different, specific annotation.

Data inconsistency describes those cases in which a sequence is nominally assigned to the orphan enzyme activity, but problems in annotation or presentation make it so that the enzymatic activity cannot be located in sequence databases. The majority of these cases (110) involved the simple absence of the EC number from a sequence that was otherwise annotated with the correct activity in NCBI Entrez Protein resource. In 28 instances the sequence had been identified with names other than any of those included in the official EC listing for the enzyme. In 3 cases, sequences had been assigned incorrect EC numbers, precluding identification with the correct numbers.

The initial orphan enzyme list used in this analysis was generated in 2009. During the time in which we did the analysis and identified 275 orphan enzyme sequences, a little more than a third of these orphans were identified in parallel by other curation efforts. The most significant contribution to this identification of sequence data came from UniProt, which continues to be at the forefront of effective and thorough manual curation of protein sequences [12].

### Sequence identification by literature search for enzyme names

We identified sequence data for putative orphan enzymes via a combination of literature and databases searches, partial sequence data, and the use of other identification information in combination with a sequenced genome.

The vast majority of sequences (over 97%) were identified by tracing the enzyme through the literature and associated databases. These enzymes were connected to their sequences using open reading frame names or other identifiers collected from publications describing the enzymes. In those cases where these identifiers could be used, individually or in combination, to unambiguously match the enzyme being reviewed to a specific gene in a sequenced genome, we assigned the enzyme activity to that specific sequence.

For example, the sequence for L-rhamnose-1-dehydrogenase (EC # 1.1.1.173) was identified via this approach. Although searches for the EC number yielded no results, a search for the enzyme activity's name in PubMed led to a 2008 publication identifying a gene catalyzing this activity in the yeast *Pichia stipitis* (now called *Scheffersomyces stipitis*) [19]. Although this paper did not contain an accession number or link to a deposited sequence, we were able to combine the name of the gene coding for L-rhamnose-1-dehydrogenase (*RHA1*) with the sequenced, annotated genome to identify the amino acid sequence for this enzyme. In this case, the new annotation of “L-rhamnose-1-dehydrogenase” replaced the prior annotation for *RHA1* of “glucose 1-dehydrogenase II.”

Sequences were also identified using complete or partial sequence data. In some cases, papers contained actual accession numbers, even though the deposited sequence was not identified as the enzyme in question. Other orphans were identified using complete amino acid sequence data contained within papers. These sequences were frequently found within figures where they had not been captured by optical character recognition (OCR) methods and thus were not machine-searchable even in electronic versions of the paper. Five orphans were identified from partial sequence data contained within papers. Most frequently these partial sequences were from amino-terminal sequencing of the protein. In those cases where the source organism or a very similar organism had been fully sequenced, the 10–20 amino acid residues revealed by amino-terminal sequencing were sufficient to uniquely identify the enzyme by comparing them to the full set of amino acid sequences for that organism, especially when other identification data such as molecular weight are available. It is important when evaluating N-terminal sequence data to account for common errors in Edman Degradation (or the other specific method that was used) such as the destruction of cysteine residues during the sequencing procedure.

Glycochenodeoxycholate sulfotransferase (EC # 2.8.2.34) was one example enzyme identified using amino-terminal sequence data. A 1989 paper describing the purification of this enzyme from rat (*Rattus norvegicus*) also included amino terminal sequencing covering the first 31–33 residues of the protein [20]. As sometimes occurs with amino-terminal sequencing data, there were two potential residue positions that were clearly ambiguous. We also proactively considered the possibility of common sequencing errors in the procedure used (Edman Degradation), such as loss of cysteine residues and de-amination of other residues. To account for these considerations we used BLAST to compare each potential variation on the amino-terminal sequence to the *R. norvegicus* genome (AAA41356.1) [1]. This identified a nearly complete match to one of the possible amino-terminal sequences, which became fully complete when we accounted for two likely sequencing errors, including the loss of a cysteine. We further fact-checked this assignment by comparing the experimentally derived molecular weight of approximately 30 kDa as described in the source publication with the calculated molecular weight of approximately 33 kDa based on the associated full amino acid sequence. Finally, identification of a conserved sulfotransferase domain (pfam00685) reinforced the assignment of the Glycochenodeoxycholate sulfotransferase activity to this sequence [21]. Given these considerations, we were confident that we had properly associated this enzyme to its full amino acid sequence.

In two cases, experimentally determined molecular weight data was sufficient, in combination with computed molecular weights based on gene translations from a fully sequence genome, to uniquely identify a sequence for the enzyme.

### Observed traits of orphan enzymes

In the previous evaluation of a sampling of 228 putative orphans, approximately 20% could be resolved via literature, database, and patent searches [6]. That percentage slight increased (to 25%) during our evaluation of the full set of 1,122 putative orphans. Given this relative consistency, it is likely that the characteristics of future putative orphan enzymes that have not yet been assigned EC numbers will match those we evaluated in the current work.

During the process of evaluating putative orphan enzymes, we noticed that, surprisingly, many orphans were characterized in relatively recent publications, contrary to our expectation that ensuring complete sequence labeling and deposition would be routine at this point. In a few other cases, orphan enzymes were commercially available, despite the lack of sequence data. Our approximate estimate from reviewing the remaining orphan enzymes is that 10–20% of the time, a protein sample may be available from the lab that originally characterized the activity (without following up with a statistically representative sampling of these laboratories, we cannot provide a more specific estimate). We have successfully followed up in several of these cases, acquiring samples from other researchers and completing the identification process via mass spectroscopy sequencing [22].

Very infrequently, amino-terminal sequence data will be available but the source organism will not have been sequenced. We identified eight such cases in the work described here (TABLE S1). Sometimes the sequence can be matched to a sequence within a genome from a very closely related organism, or can be used to identify a handful of candidate sequences for subsequent assay. In many cases, the original source organism is lost (a sample was not retained in any strain collections) or is effectively lost (e.g. unhelpfully identified as “a *Pseudomonas* species”).

## Discussion

When we began this work, we identified 1,122 putative orphan enzymes out of 4,858 enzymes then classified within the EC system (approximately 23%). We subsequently located sequences for approximately 25% (275) of these orphans via searches of the literature, databases, and patents.

Based on this experience, we developed a clarified and refined process for evaluating orphan enzymes with the goals of finding sequence data when possible and collecting identification information to support computational or laboratory identification of orphans otherwise. We now have a better picture of the likely outcomes when a new orphan enzyme is encountered, which can help researchers determine the most efficient approach for each orphan. These findings also factor into our understanding of how we can avoid generating new orphan enzymes and speed the overall process of finding sequences for known orphans.

### Refining the orphan enzyme evaluation process

**Figure 4:**
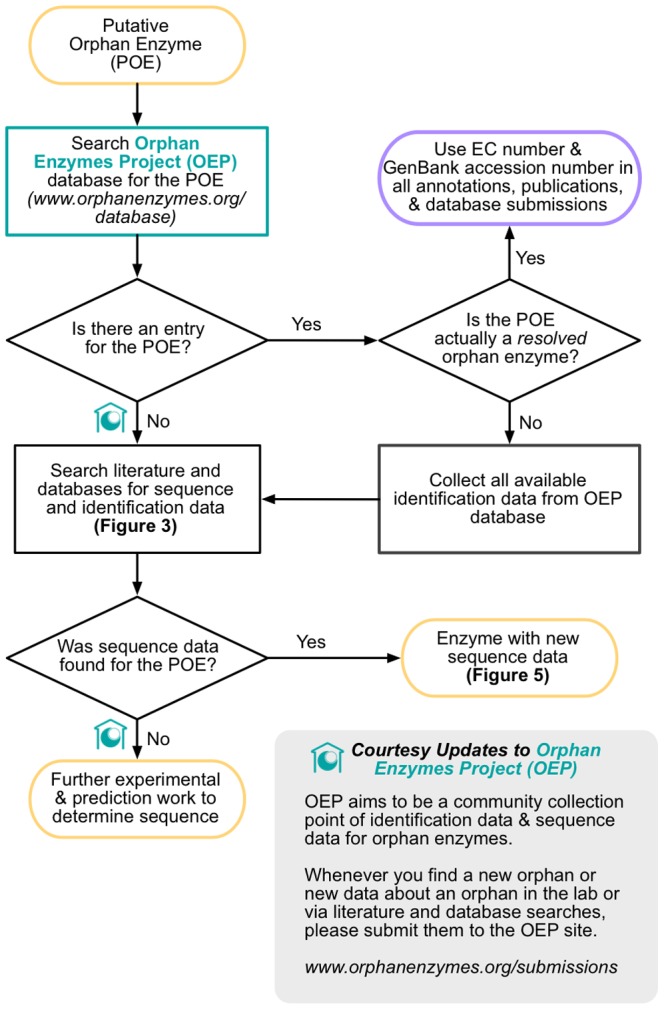
The end-to-end process of resolving an orphan enzyme may include literature searches, database searches, and laboratory work. Beginning with a putative orphan enzyme (POE) (yellow), an investigator can maximize the likelihood of finding sequence data while minimizing effort by following a few steps. An immediate search of the OEP database will indicate if the orphan is already recognized as such and give the researcher access to any data about that orphan enzyme that others have already collected, including if it has been resolved (and perhaps the link between sequence and activity simply haven't been propagated to major sequence databases yet). The next steps are to carry out a literature and database evaluation of the orphan and then potentially follow that with laboratory identification. It may be helpful to submit information about the orphan enzyme, including the fact that it exists as well as any supporting identification information, to the OEP web site at the two marked points in the process (OEP symbols). This makes the information available to others in the research community who may be able to help identify sequence for the enzyme activity.

We have developed a refined approach to evaluating future orphans based on our experience evaluating 1,122 putative orphan enzymes (FIGURE 4).

The goal for the orphan enzyme evaluation process is to rapidly determine whether sequence data is available, and just as quickly collect identification information when it is not. To do this, we want to leverage the data collection efforts of prior researchers whenever possible. We recommend that a researcher starting with a new putative orphan enzyme first check the Orphan Enzymes Project (OEP) website (www.orphanenzymes.org) to discover if the orphan has already been recognized and work has been carried out to resolve it. In some cases, sequence may have been identified and sequence databases have simply not yet been updated. In most cases, a known orphan will be listed with any associated identification information and relevant citations. If there is no listing for the new orphan enzyme, it can be submitted to the OEP database. This alerts researchers to an outstanding orphan, and serves as a collection point for identification information.

The literature and database search follows next. Although we expect this method to find sequence data only 20% of the time, the cost of doing the search is so low that it is always recommended. In addition, even if sequence is not found, this search process helps collect identification information that is critical to being able to either validate computational predictions or purify, assay, and sequence the orphan enzyme in the lab.

We recommend carrying out the literature and database evaluation essentially as described above (FIGURE 3). The first step should always be a thorough name and synonym search of major databases such as UniProt, NCBI Entrez Protein database, and BRENDA [12], [17]. As we described in Results, the UniProt Consortium identified numerous sequences for orphan enzymes in parallel with our efforts. It is always possible that an orphan has been identified by UniProt or other curation teams in the time between its identification by a researcher and when that researcher attempts to resolve it. It is often helpful to search these resources using incomplete or partial versions of an enzyme activity's names. Of the “mismatched” names we saw in our own search, the most frequent were those in which elements in the enzyme activity name describing the substrate or product were dropped (e.g. “fluorenol dehydrogenase” instead of “fluoren-9-ol dehydrogenase”). Permuting and modifying the names used in searches adds a little effort up front but can save considerable time in the long run by turning up sequence or identification information that might otherwise have been missed.

There are likely to be many orphan enzymes that do not currently have assigned EC numbers. In this case, the recommendations are essentially the same, with the caveat that the BRENDA resource will not apply. Otherwise, it is still entirely feasible to search UniProt, NCBI Entrez Proteins, and other online resources using the known names and synonyms for the activity, exactly as we describe in our search process. One concern in stepping outside of the EC system is name ambiguity. The EC system relieves some of the burden of name ambiguity by providing a curated list of defined enzyme activities that have been reviewed and deemed unique. This resource is not available for enzymes that have not yet been classified. Continuing advances in resolving protein and gene name ambiguities should reduce this problem in the future [23], [24].

In addition to checking the OEP database and potentially submitting a new orphan enzyme, we also recommend updating the OEP database with the results of any literature evaluation and later experimental identification efforts. We are currently developing the OEP database to collect data on publications, identification information, and other community notes on each remaining orphan enzyme activity. Even if no sequence data are found for an orphan, collecting all of the available citations and identification information in one place helps other researchers who may be interested in resolving the orphan, provides a ready resource for database curators who are incorporating information about the enzyme activity, and can serve as a starting point for collaborations. For example, a purification, assay, or sequencing process that is too costly or difficult for one research group may fit the expertise of another group, allowing a fruitful, low-cost solution via collaboration.

### Most orphans will still need to be identified in the lab

Our results suggest that any newly discovered orphan enzymes will likely need to be identified in the lab. A majority of putative orphans (75%) can be expected to genuinely lack associated sequence data. These enzymes will need to be resolved by *de novo* purification of the enzyme from its source, by sequencing from retained samples of the enzyme, or by computationally predicting candidate sequences and then validating the predictions in the lab. All three methods rely on collecting those publications that describe the original characterization, purification, and assay of the enzyme activity being studied. It will also be necessary to develop methods to automate the updating, troubleshooting, and running of purification and assay protocols to improve throughput. In our experience, the time and effort involved in troubleshooting and updating protocols one at a time is prohibitive.

Within the 25% of putative orphans for which sequence data is available, the vast majority will be identifiable using databases and the literature. Although it remains worthwhile to keep an eye out for amino-terminal sequence data and other identifying characteristics, we expect that they will only rarely be used to identify orphan enzyme sequences in those cases where the enzyme has been fully sequenced.

### How the overall orphan enzyme problem can be resolved

We expect that more orphan enzymes await discovery outside the bounds of the current EC system. Individual orphan enzymes can be found by researchers reviewing the literature as part of hypothesis generation or, increasingly, in the course of developing synthetic biology solutions that hinge on enzyme activities that have not been studied previously [25]. Database curators are also likely to be a rich source of new putative orphans, especially as curators at current model organism databases work their way through the specialized literature for their organism. In fact, since EC numbers are being actively assigned to enzymes that were characterized well before the age of high throughput sequencing, many newly assigned EC activities lack sequence information and thus are orphans. There are likely to also be more “genuinely new” orphans on occasion, as researchers who are actively characterizing enzymes hit roadblocks in the purification or sequencing processes. As we have seen in our associated laboratory research, many researchers who are experts in a non-enzymatic field of study can be stymied by irregular or “tricky” purification or sequencing [22].

### How do we avoid generating more orphan enzymes?

As we saw in our evaluation of newly resolved orphans, a significant portion of putative orphan enzymes were “orphaned” despite the presence of sequence information. This was especially prevalent as a source of more recent orphan enzymes, as it is now rare for an enzyme to be experimentally characterized but not sequenced. In fact, the sequencing step is only the first of three chokepoints where an orphan enzyme can be generated (FIGURE 2).

**Figure 5:**
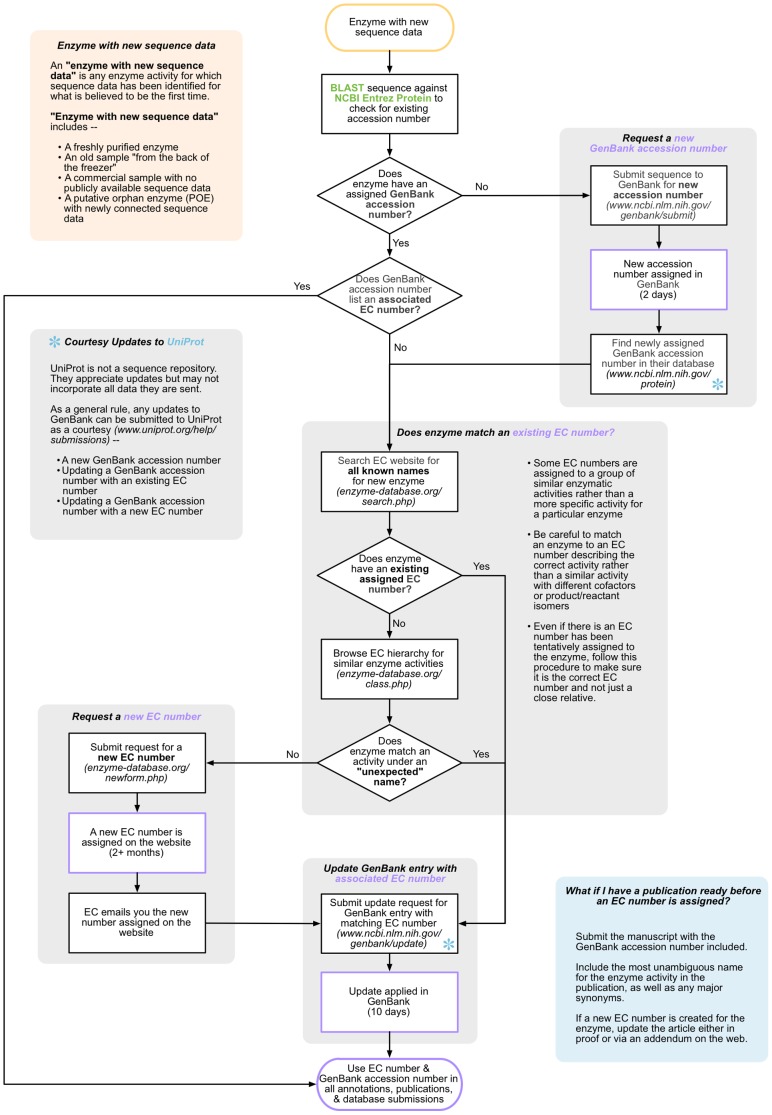
Orphan creation can be avoided by ensuring each enzyme has an accession number and an EC number. Starting with an enzyme with new sequence data (yellow and linked box), there is a well-defined set of steps to follow to avoid generating new orphan enzymes. Following this process ensures that each enzyme will be properly linked to a GenBank accession number and whenever possible, an EC number. It also helps prevent assigning incorrect EC numbers or not assigning an EC number when one that fits the enzyme activity is already available. Since the process of requesting and generating a new EC number can take 2+ months, papers may need to be submitted before an EC number exists and then the new EC number added to them in proof or via addenda (blue box). We recommend as a courtesy that researchers also update UniProt (blue asterisk) at any point at which the GenBank record for an enzyme is updated. Sequences and requests can be submitted to both GenBank and UniProt regardless of whether the data involved has been published in the peer-reviewed literature. Once an enzyme has both an associated accession number and EC number, these should be used in all future annotations, publications, and database submissions related to the enzyme.

Based on our examination of orphan enzymes and their causes, we have put together a set of “best practices” for handling any enzyme with new sequence data (FIGURE 5). The goal of this step-by-step guide is to ensure that each enzyme sequence is properly deposited in a major sequence resource with an accession number and an EC number. This helps to unambiguously link sequence and function, which in turn is necessary for genome annotation, sequence-based prediction tools, and a host of other applications (FIGURE 1).

The first major step is acquiring a GenBank accession number. For a newly sequenced enzyme, this usually involves depositing the sequence as well, although it is important to carry out a BLAST search first to catch the increasingly common case in which the enzyme was previously sequenced and the sequence was not assigned the enzymatic function. Sequence data can also be deposited in the DNA Data Bank of Japan (DDBJ) or the European Nucleotide Archive (ENA). All three databases exchange data daily as part of the International Nucleotide Sequence Database Collaboration (INSDC), so depositing sequence ensures it will be propagate to all three.

The second big step is to ensure that the enzyme is associated with an EC number. It is important to both search and browse the EC hierarchy at this step to make sure that if there is a matching existing EC number, it is associated with the enzyme. However, it is similarly important to ensure that an enzyme is not incorrectly matched to an EC number that describes a similar, but not exactly matched, activity. In reviewing orphan enzymes, we encountered many instances of enzymes that were cross-annotated to a very similar activity with an alternate EC number. There are many activities in the EC system that carry out the same chemical transformation but use a different cofactor (e.g. NADH versus NADPH) or that generate a different product isomer. In these cases, enzymes are sometimes annotated with the EC number of the opposite cofactor or isomer variant, leading to the dual problems of incorrect annotation of the enzyme and the continued incorrect “orphan” status of the other isomer. An informal evaluation of example cases where sequences are known for all cofactor variants shows that they are often highly similar. Since these cofactor variants can have such similar sequences, we believe that in many cases sequences that should be assigned to the orphan enzyme (an ortholog of unknown sequence) are instead assigned to its highly similar, cofactor-varying paralog.

For example, mevaldate reductase (EC 1.1.1.32) catalyzes the conversion of mevalonate to mevaldate with NAD+ as the cofactor [26]. This enzyme activity is not an orphan, and thus new sequences that are similar to those annotated as mevaldate reductase are likely to be assigned this EC number as well. However, there is also mevaldate reductase (NADPH) (EC 1.1.1.33) which catalyzes the same reaction, using NADP+ as a cofactor [27]. As an orphan enzyme activity, this EC will never be assigned to any sequences, and may erroneously be replaced by EC 1.1.1.32. Cofactor variations such as this can lead directly to problems in metabolic predictions and other computational and experimental analyses of an organism's biology.

This kind of “rich get richer” situation in annotation is subtle, but may be wide-ranging and deserves further study. It is possible that many cofactor or other specificity constraints may be widely mis-annotated due to over-annotation to the one known variation of the activity.

Unique identifiers such as EC numbers help ensure that specific chemical transformations (specific sets of substrates and products) that are catalyzed by enzymes can be unambiguously tied to sequence data. This kind of unambiguous linkage helps prevent problems such as inferring the presence of the wrong metabolic pathway or other biological system based on free text genome annotations that can be interpreted to mean more than one variant in a set of highly similar activities [28]–[30]. As described above, a common problem in making metabolic predictions based on annotations is the lack of specificity about the product isomer generated by a given enzyme. At a minimum, having unique identifiers for specific enzyme activities cuts down on wasted time in reviewing the literature on an enzyme of interest, much in the same way that PubMed IDs or DOIs help point readers to the exact article they need, Gene Ontology terms link the same gene functions and roles across multiple organisms, and efforts such as ORCID (http://www.orcid.org) help point researchers to the correct scientific author [31], [32].

The assignment of an EC number requires submission to the EC, followed by internal review within the EC and then a 2-month external community review process. As a consequence, there may be occasions where publications concerning an enzyme are prepared for submission before a decision has been made on assigning an EC number. In these cases, we encourage researchers to submit their publications using the most unambiguous name for the enzyme they can, as well as several synonyms, and then to later update publications either in proof or via an addendum to include the EC number. Researchers are also encouraged to state in their sequence submissions and publications that the enzyme was submitted to the EC under a given name and on a given date.

We also recommend that UniProt and other sequence databases be updated, as a courtesy, each time an enzyme's GenBank or similar record undergoes a significant update (such as initial sequence deposition, or assignment of an EC number).

### Closing the gaps

These “best practices” should prevent creation of new orphan enzymes. However, it also highlights why the gaps that generate new orphan enzymes exist (FIGURE 2). The turnaround time for sequence deposition and updates in GenBank is rapid (2 days and 10 days, respectively). However, there is no system in place to alert the researcher that their accession has been assigned or that their update has been accepted. Updating materials with the new accession number thus requires at least two steps by the researcher, first to check on their sequence's status in GenBank and then to update a manuscript, submitted paper, or other material with the new accession number. A researcher would then have to repeat the process of updating the GenBank entry once an EC number is assigned, and then return to publications and other materials to update them with that newly assigned EC number. The incentive is often absent to take these extra steps, and with graduate students, postdocs, and other researchers moving on from a research group many of these manual updates simply will not happen. An alternate approach is to seek the assignment of an EC number first and submit that EC number with the initial GenBank submission. However, the differing timeframes for submitting a sequence and having an EC suggestion evaluated mean that it can be difficult to have an EC number in place before a sequence is submitted.

The easiest way to work around these potential gaps is to provide explicit links between entries. One possible approach would be to create an enzyme activity deposition identifier (analogous to GenBank identifiers) that is assigned to a proposed novel enzymatic activity while it awaits review by the Enzyme Commission. This identifier would then be used in place of an EC number in GenBank submissions and publications and be tracked in the EC submission. This identifier could then be replaced with the final approved EC number in each of these cases retrospectively. One option would be for the EC or www.orphanenzymes.org to issue globally-unique identifiers and then orchestrate the update process of swapping the EC number for this identifier in cooperation with the EC, GenBank, and journals. In fact, the Enzyme Commission already issues “temporary EC numbers” to submissions, while they await rigorous review [33], so the easiest solution may be to merely issue temporary EC numbers automatically using an online ticketing system. To make this genuinely automatic would require the cooperation of these resources to retrieve identifier mapping data from the EC or OEP and then update their own records. There is a survey posted at www.orphanenzymes.org to gather community opinion on how this solution could be enacted. We encourage interested researchers to complete the survey to help decide how best to resolve this problem.

## Methods

### Definitions

An “orphan enzyme” is an enzyme where the biochemical activity has been experimentally characterized, has substrates and products that are distinct from other characterized enzymes, and has no associated sequence data in any major sequence databases.

A “putative orphan enzyme” is an orphan enzyme prior to our search of the literature and sequence databases.

A “true orphan enzyme” is an orphan enzyme for which the absence of sequence data from major sequence databases has been confirmed by a search of the literature and databases.

### Generating the starting list of putative orphan enzyme activities

We generated the initial list of putative orphan enzyme activities by combining the results of a series of database queries and searches carried out in March and April 2009.

A draft putative orphan list was assembled by searching for EC numbers that lacked associated amino acid or protein sequence data in each of Enzyme DB, SwissProt, TrEMBL, BioCyc proteins, BioCyc reactions, and Orenza. In March 2009 we performed a number of SQL queries against bioinformatics databases using the BioWarehouse biological database warehousing system [34]. The objectives of the queries were to create a definitive list of all defined EC numbers and create a list of EC numbers with known associated sequence (which were thus ruled out from being orphan enzymes). The BioWarehouse instance was loaded with recent versions of the following resources: ENZYME, MetaCyc, the BioCyc database collection, SwissProt, and TrEMBL [12]–[14]. Using the ENZYME database as our definitive list of all EC numbers, we searched ENZYME and the other databases for any linkages between EC numbers and sequence data. Each EC number for which we could not find evidence of associated sequence data was retained on a list of putative orphan enzymes.

We further cross-checked our list of putative orphan enzymes against the Orenza database for cases in which the Orenza project had been able to resolve some of the orphans [15].

In April 2009 we expanded our search to include resources at NCBI and the BRENDA database [17], [35]. Both of these searches were conducted using our working list of putative orphan enzymes. A set of shell scripts were created to automate querying of both NCBI and BRENDA for sequence data using search form URLS from their respective websites. Searches were carried out using the accepted, systematic, and other names for each putative orphan enzyme activity. We evaluated the sequences that were identified via this set of searches and discovered that the majority of them were incorrectly assigned to orphan EC numbers due to three instances of systematic, incorrect EC assignments originating in one specific genome sequencing center (this systematic error was reported back to the sequencing center). These incorrect pairings of EC numbers with sequences were discarded. The remaining search results slightly reduced the total count in the draft putative orphan list described above.

We also used Gene Ontology (GO) mappings between the database resources described above in an effort to link sequences to putative orphan activities [32]. We discovered no additional sequences with that approach.

Four putative orphans were removed during the study period due to deletion from the official EC hierarchy.

This overall approach yielded the final starting list of 1,122 putative orphans (TABLE S1).

### Evaluating putative orphan enzyme activities

Each putative orphan enzyme was evaluated using the same series of operations.

First, the reviewer collected the accepted name, systematic name, and other names from the orphan enzyme's reference page at the IUBMB Enzyme Nomenclature site.

The reviewer then checked first the BRENDA and then the MetaCyc databases for the EC activity. In some cases, the BRENDA or MetaCyc resource contained links to sequence data (in the “Cloned/COMMENTARY” section on BRENDA and in the protein page on MetaCyc) that had not been amenable to automated searches and which did not appear in the major annotation databases. If sequence data was obtained at this point, the sequence or associated accession numbers were collected and the search was stopped. Citations associated with demonstrating the enzyme's activity, purification, or cloning were collected from BRENDA and MetaCyc.

The reviewer collected additional citations by searching PubMed with the EC number and the names for the orphan activity (searching each name individually). Reviewers searched with permutations on the orphan activity's name as well. Standard permutations included removing numbers, dashes, and references to specific cofactors or substrate or product chirality.

For each citation, the reviewer checked the actual publication for sequence data whenever possible. Some research publications were not accessible, most often due to lack of digital archiving of low circulation or out-of-print journals. In some of these cases, reviewers relied on curated data contained within BRENDA or MetaCyc for the identification information collection process.

For each publication, the reviewer collected the following whenever possible:

1. Source organism(s)
2. Whether or not a purification method was described
3. Whether or not an assay method was described
4. Sequence (including accession numbers leading to sequence and partial sequence data)
5. Molecular weight(s)
6. Isoelectric point(s)
7. Whether kinetics data were described
8. Notes on any other area of interest (e.g. extreme lability of the enzyme)

Data were collected in the associated data file with one line for each combination of EC, organism, and sequence (TABLE S2).

Following literature analysis, reviewers examined patents for possible sequence data. This was performed by searching against the United States Patent and Trademark Office database using the EC number and collected names (searched individually) for the orphan enzyme [18].

Each EC activity review was stopped when at least one complete amino acid sequence had been identified. We did not require that this sequence come from one of the source organisms attested to in the EC description for the activity, as EC numbers are assigned solely based on the reaction catalyzed, irrespective of source organism. In a few cases, we continued to search after a single sequence had been found, due to published experimental characterization that indicated that the activity was catalyzed by a multimer comprising distinct protein subunits.

### Finding sequences for orphans

As described above, the search process collected accession numbers as well as full and partial amino acid and genetic sequence.

Whenever possible, sequences were associated with an accession number from the NCBI Entrez Protein resource.

When the review uncovered full or significant partial sequence data, it was evaluated by providing the sequence data as the query sequence in a BLAST search against the NCBI Non-Redundant amino acid sequence database (nrdb) [35]. In every case where the genome sequence of the source organism was available, this led to a perfect match between at least one variant of the full or partial sequence and a sequence segment of the same length in a protein from the source organism.

BLAST searches were carried out using blastp (protein-protein BLAST) against the Non-Redundant protein sequences database (nr db) via the NCBI Blast web site using the BLOSUM matrices. For amino-terminal partial sequence data, the search parameters were adjusted based on the automated suggestion from the NCBI BLAST web site (adjusted parameters were: expect threshold  = 200,000, word size  = 2, PAM30 matrix, no compositional adjustments).

## Supporting Information

Table S1 All evaluated enzymes with final status and sequence data. This table includes the final status for each enzyme evaluated in this study. For each enzyme, the table lists its EC number, enzyme name, status (“resolved” or “open”), sequence (either an accession number or an amino-acid sequence), and notes. Notes in this table are limited to discussion of whether N-terminal sequences are available and possible identity with other EC numbers.
(XLS)

Table S2 All identification information collected in this study. This table includes all the identification information collected in this study, for both open and resolved orphan enzymes. Each row lists an EC number followed by the source document for the information in the rest of the row (usually a PubMed ID number, but sometimes a free text citation). Other information includes the source organism in which the activity was identified in that publication, whether the publication describes the enzyme activity's purification and assay, sequence information (either an accession number or a full or partial amino-acid sequence), molecular weight (in kiloDaltons), isoeletric point, kinetics, and any additional notes.
(XLS)
